# Investigating the relationship of indoor temperature and humidity with sleeping quality in private residential care homes for persons with disabilities in Hong Kong

**DOI:** 10.3389/fpubh.2026.1748619

**Published:** 2026-02-23

**Authors:** Ho Lim Lee, Ngai Sze Wong

**Affiliations:** 1 Professional Outreaching Team for Private Residential Care Homes for Persons with Disabilities (Team 4), Christian Family Service Centre, Hong Kong, Hong Kong SAR, China; 2 S. H. Ho Research Centre for Infectious Diseases, JC School of Public Health and Primary Care, Faculty of Medicine, CUHK, Hong Kong, Hong Kong SAR, China

**Keywords:** indoor temperature, persons with disabilities, relative humidity, residential care homes, sleep quality

## Abstract

**Objective:**

This study investigated the relationship between indoor temperature, humidity, and sleep quality in private residential care homes for persons with disabilities (PRCHDs) in Hong Kong, a vulnerable and under-researched population.

**Methods:**

A 6 month pilot study (August 2024–February 2025) was conducted in three PRCHDs. Smart sensors monitored indoor temperature and relative humidity in dining areas and bedrooms. Thirty residents with disabilities residing in PRCHDs completed four surveys assessing sleep quality using the Pittsburgh Sleep Quality Index (PSQI). Time-series analysis compared indoor conditions with outdoor data, while statistical models analyzed associations between PSQI scores and environmental factors.

**Results:**

Many participants residing in experienced poor sleep quality (PSQI > 5), especially in summer (30–73%). Indoor temperatures in PRCHDs often exceeded outdoor levels, particularly during warmer months. Significant associations were observed between sleep quality and indoor temperature, relative humidity, sleeping periods, and self-perceived humidity (*p* < 0.05).

**Conclusion:**

Findings highlight the need to improve environmental conditions in PRCHDs, particularly during summer. Recommendations include improving ventilation, increasing air conditioning use, and promoting outdoor activities to enhance sleep quality and overall well-being.

## Introduction

Relative humidity and indoor temperature are linked to several detrimental health effects throughout the year. Studies showed that exposure to heat is linked to a variety of diseases and symptoms, including pain, heat exhaustion, and heat stroke (1–5). Several studies and reviews have indicated that heat exacerbates pre-existing conditions, such as diabetes, asthma, cerebrovascular, respiratory, and cardiovascular illnesses (1, 3–5). Additionally, although high humidity does not directly affect people’s physical capacity, it facilitates the growth and spread of biotic agents, which can result in respiratory discomfort and allergies, and is recognized as a primary health effect (6).

The connection between environmental factors and mental health has also long been recognized, and it has gained more attention due to climate change recently (7–10). High ambient temperatures are associated with various mental health outcomes, including stress, suicidal ideation, exacerbation of schizophrenia, anxiety, and depression (10–15). In addition, high relative humidity was suggested to amplify the effect of heat on distress levels for people with depression or anxiety (16, 17). Increasing hospital admissions for schizophrenia are also related to high temperatures, according to studies in 2019 and 2024, conducted in China and Canada, respectively (18, 19). According to a long-term study, symptoms such as headaches, cramps, thirst, and exhaustion grow in direct proportion to temperature increases, particularly between 30 and 33 °C (20). Temperature also plays a crucial role in regulating sleep, the quality of which is known to be related to mental health, including depression, anxiety, rumination, stress, and psychosis symptoms (21–23). Studies have shown that sleeping quality can be affected by ambient temperature, with a significant drop in sleep quality observed when the temperature rises above 29 °C (24–26). A systematic review showed some data suggesting that excessive environmental heat cannot only exacerbate symptoms of both dementia and schizophrenia, but also that these populations are more temperature sensitive (27). According to estimates from the World Health Organization, the prevalence of lifetime mental disorders is estimated to range from 18.1 to 36.1%, which includes mood disorders and schizophrenia (28). This large proportion of the population, therefore, needs to be more concerned in the future years.

In Hong Kong, time-series studies showed similar results that higher hospital physical and mental-related admission rates were associated with high temperatures (29, 30). With an annual mean temperature of 24.5 °C, 2023 was among the second-warmest years ever recorded. Specifically, the summertime mean temperature (June–August) hit a record-breaking 29.7 °C (31). Due to climate change, global temperatures are expected to continue rising.

Residential care homes for persons with disabilities (RCHDs) are one of the places for persons with disabilities, who may suffer from physical or mental disability, to live. Most of the private RCHDs (PRCHDs) are located in old buildings in hustle areas (32), and air conditioning systems and ventilation are not always satisfactory. Due to physical or mental health issues, most of the residents spend much of their time indoors, which is easily affected by indoor temperature and humidity changes. However, as the PRCHDs are private properties, in-depth research is not commonly allowed.

This study aims to investigate the indoor temperature and humidity in PRCHDs in Hong Kong, including: (1) To measure the indoor temperature and humidity at PRCHDs longitudinally from summer to winter; (2) To investigate the association between indoor temperature and relative humidity at PRCHDs and the sleeping quality of care home residents with disabilities.

## Methods

### Study design

This is a 6 month longitudinal study with primary data collection on environmental and sleep quality. There are 25 PRCHDs associated with the Christian Family Service Centre (CFSC) in Hong Kong. Since the PRCHDs are private premises, a total of 3 PRCHDs agreed to participate in this study. The in-charge person of each participating PRCHD was contacted face-to-face for permission to place HT1 Temperature and Humidity Smart Sensors. The content, including the study objective, methodology, and duration, was explained with written consent.

Inclusion criteria included care home residents with disabilities aged 18 or above. Those who were unable to respond to face-to-face surveys owing to cognitive or physical disability were excluded. The study design, including the study objective, survey content, and duration, was clearly explained in the written consent before the study. Informed consent was obtained from all related residents. Those who were literate signed the consent forms themselves, and the illiterate had a family member sign on their behalf. In a few cases with no family member who could sign, the local physician signed to witness the subject’s consent. All the study’s methods conformed to any relevant guidelines and regulations.

### Data collection

A general description of the physical environment of all participating PRCHDs was recorded, including information such as total area, ventilation conditions, and air-conditioning systems. The HT1 Temperature and Humidity Smart Sensor was employed in this study to measure the indoor temperature and relative humidity (RH). It has a real-time temperature and RH measurement range of −40 °C to 60 °C and 0 to 100%, respectively. The sensor has an accuracy of ±0.3 °C for temperature and ±3% for RH. The time resolution was set to 1 min.

From late August 2024 to February 2025, three smart sensors, one in the dining area and 2 in the bedrooms (consent of residents in the bedrooms obtained) of each PRCHD were placed, as they were the areas that participants spent most of their time. The sensors were placed around 1–1.5 m above the ground to simulate the residents’ sitting position and bed height. All sensors were placed far from direct sunlight and ventilation sources, to prevent affecting the results. Although noise levels, lighting conditions, nighttime activities, and room crowding are all plausible determinants of sleep and may act as confounders, they were not collected due to resource constraints and the exploratory nature of the study.

Four rounds of face-to-face surveys were conducted in the participant’s bedroom in late September, October, November 2024, and January 2025, and no data collection was scheduled in December 2024. The timeframe was selected to approximate warm, transitional, and cooler conditions. As to the definition of the timeframe, we stated that Hong Kong’s “hot/warm” season during the study corresponded mainly to late August to October; “transitional” or “neutral” conditions around November; and “cool/cold” conditions in December to February. This classification was based on descriptive terms of the Hong Kong Observatory, with Cool: 13–17 °C, Mild: 18–22 °C, Warm: 23–27 °C (33). Also, while the Hong Kong Observatory defined summer in Hong Kong as June, July, and August, our study included September and October as the summer season, given the relatively high temperatures and delayed consent from the in-charge persons, which limited the data collection period. All surveys were conducted at the end of the corresponding month, within 3 days for all participants residing in PRCHDs, to ensure the similarity of environmental conditions. The survey was conducted in around 20 min, with the use of a structured questionnaire (Supplementary Questionnaire S1). Basic demographic data such as age, use of sleeping-related medication, and physical, medical, and mental diagnoses were collected. Additionally, questions related to residents’ everyday routine were asked further to investigate the association between environmental change and sleep quality. For example, time (hours) spent in the dining area and bedroom every day, and weekly frequency and duration of outdoor activity. The quality of their sleep was assessed with the PSQI.

The 18-item PSQI is a self-reported tool assessing overall sleep quality that was initially used to evaluate people aged 24–83 (34). There are 4 open-ended questions, and 14 items are graded on a 4-point scale. A total of 7 elements comprised the entire set of 18 items, including subjective sleep quality, latency, duration, efficiency, disturbances, use of sleep medication, and daytime dysfunction. Better sleep quality is indicated by a lower PSQI score and vice versa. Developers suggested a cutoff score of 5 for the global scale as it correctly identified 88.5% of the patient group in their validation study (34). For participants residing in PRCHDs with intellectual disabilities, additional verbal explanation with simplified language was provided to ensure their understanding. The interviewer read each PSQI question using the Chinese wording and, when comprehension difficulties arose, provided brief clarifications or paraphrases that preserved the original meaning, such as rephrasing “sleep disturbance” as “things that wake you up or make it harder to stay asleep”. The response options and scoring categories were not modified, and the interviewer avoided leading prompts or reinterpretations that could change the construct being measured. As all interviews were conducted face-to-face by the same professional, it enhanced consistency and reduced interviewer-related variability. The collected PSQI scores were linked to indoor temperature and humidity using summary statistics derived for periods corresponding to the month preceding each assessment, including the mean/maximum/minimum temperature and humidity of the relevant month.

### Data analysis

To investigate the similarity between PRCHDs indoor temperature and RH and the outdoor environment, line graphs were used to visualize time series data regarding the daily temperature and RH for both the PRCHDs and the observatory. Outdoor data can be gathered from the Hong Kong Observatory’s Open data, which updates monthly (35).

Repeated Measures ANOVA was used to examine the means of PSQI scores across the four different time points for the same 30 participants. The Kruskal-Wallis test was used for variables not following a normal distribution. The association between PSQI scores, indoor temperature, relative humidity, and other factors was analyzed by Generalized Estimating Equations (GEE) (36), which is particularly suitable for analyzing correlated data, such as repeated measures from the same participants over time in this study. The unstructured working correlation matrix was selected because it allows flexibility in estimating correlations among repeated measures without making strong assumptions about their structure. This was particularly appropriate given the exploratory nature of the study and the potential variability in environmental conditions and sleep quality across time points. Although our sample size was modest (30 participants, 4 time points), GEE remains a commonly used and robust approach for longitudinal data with correlated outcomes. To address model stability and potential multicollinearity, we initially fitted models including month and conceptually grouped predictors, then retained only variables that were statistically significant and clinically meaningful in the final model, thereby reducing the number of parameters relative to the available observations. During this process, we monitored changes in coefficient signs and magnitudes; no instability or implausible shifts in parameter estimates were observed when adding or removing predictors, suggesting that multicollinearity did not materially distort our estimates. SPSS 27.0 (SPSS Inc., Chicago, IL, United States) for Windows was used for statistical analysis.

### Ethical considerations

All collected data, including environmental information and face-to-face survey results, were kept confidential. The names of the PRCHDs and participants were encrypted and kept private, and all encrypted data was securely stored in the student’s computer, with access restricted to the student and his supervisor only. All gathered information will be permanently erased once the study is completed. No interventions were applied in the study, and the evaluation was meant to be non-invasive. Consequently, there was no additional harm or risk posed to the participants. Approval from the Survey and Behavioral Research Ethics (SBRE) was obtained for this study (Ref No: 020-24). Written consent was sought before the survey. All participants could withdraw at any time.

## Results

The basic information and environmental characteristics of the 3 PRCHDs involved in the research were collected, including the number of ventilation and air-conditioning systems (Supplementary Table S1). All PRCHDs were installed with fans and air-conditioners, operating in different periods. There were 163 residents, aged 19 to 72, living in the 3 PRCHDs.

### Temporal change of the indoor and outdoor environment

**Figure 1:**
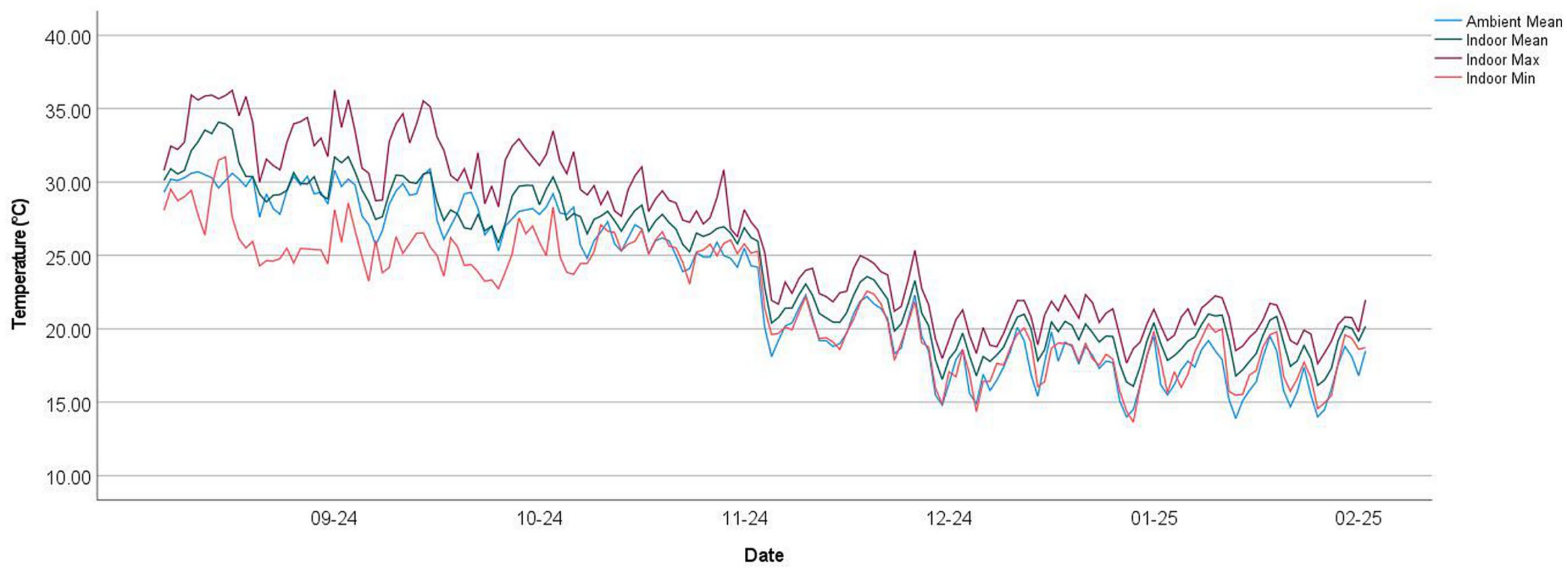
Indoor and ambient temperature in bedroom 1 of private residential care home for persons with disabilities (PRCHD) 3 from 8/2024 to 2/2025, Hong Kong. Mean, min, and max represent the daily mean, minimum, and maximum temperature, respectively.

**Figure 2:**
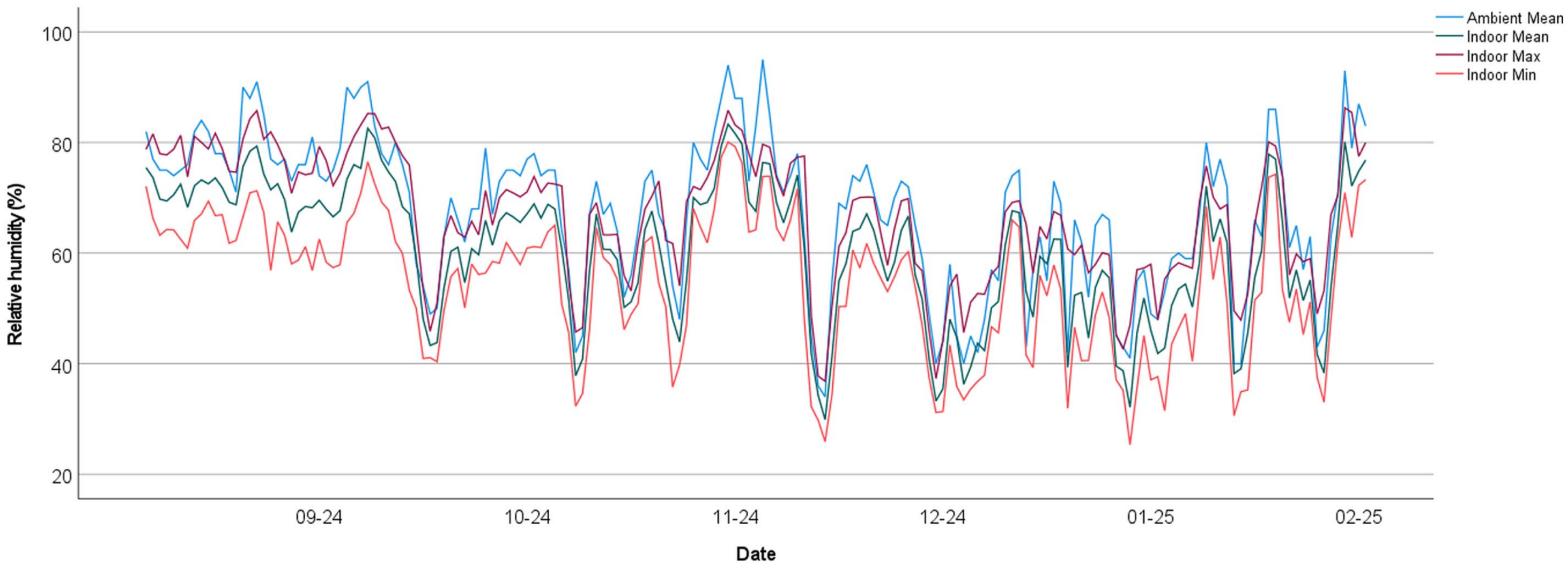
Indoor and ambient relative humidity in bedroom 1 of private residential care home for (PRCHD) 3 from 8/2024 to 2/2025, Hong Kong. Mean, min, and max represent the daily mean, minimum, and maximum relative humidity, respectively.

Indoor temperatures and relative humidity were monitored consecutively from 23/8/2024 to 15/2/2025. Maximum indoor temperatures were remarkably higher than those in outdoor environments, especially during the summer from August to mid-November, as shown in bedroom 1 of PRCHD 3 (Figures 1, 2). Mean indoor temperatures were relatively consistent with outdoor temperatures in summer, while they usually got higher than outdoors after mid-October. Minimum indoor temperatures fluctuated between different PRCHDs and rooms in summer, which followed the mean of outdoor temperatures roughly, and they remained higher than outdoor temperatures in winter. Considering sleep quality would be greatly decreased once the ambient temperature rises above 29 °C (24), the number of days in the study period over 29 °C was more than 40% for maximum (41.81–49.72%), minimum (6.21–40.68%) or mean (27.12–44.63%) values throughout among all hotels’ rooms, compared with only 23.73% for ambient mean temperature. Daily temperature fluctuations, also known as diurnal temperature variation, were significant and more pronounced in summer, as indicated by the range between maximum and minimum temperatures. Indoor RH was usually and steadily lower than outdoors, especially in winter. Results of other PRCHDs and rooms are also recorded, which showed similar results (Supplementary Figures S1, S2).

### General characteristics of participants and their sleep quality

A total of 30 participants residing in PRCHDs completed 4 face-to-face surveys and PSQI assessments (Table 1). The average age of them was 49.5 years, ranging from 18 to 71 years, with a standard deviation of 12.6. Most participants were male (60%). Sixty percent of them had mental illness, with a record of 53.3% using antipsychotics regularly. Most of the participants had a regular and stable pattern of antipsychotic drug use and outgoing behavior in the study period.

**Table 1:** Demographic characteristics of participants residing in private residential care homes for persons with disabilities (PRCHDs), Hong Kong, 2024–2025.

Characteristics	Total (*n* = 30) (%)	PRCHD 1 (*n* = 10)	PRCHD 2 (*n* = 10)	PRCHD 3 (*n* = 10)
Gender				
Female	12 (40%)	7	3	2
Male	18 (60%)	3	7	8
Age				
0–20 years	1 (3.33%)	0	0	1
21–40 years	5 (16.67%)	2	0	3
41–60 years	20 (66.67%)	6	10	4
≥61 years	4 (13.3%)	2	0	2
Major diagnosis				
Stroke	3 (10%)	2	1	0
Intellectual disability	9 (30%)	2	0	7
Mental illness	18 (60%)	6	9	3
Use of antipsychotic				
Use of antipsychotic	16 (53.33%)	6	7	3
No use of antipsychotic	14 (46.67%)	4	3	7

**Figure 3:**
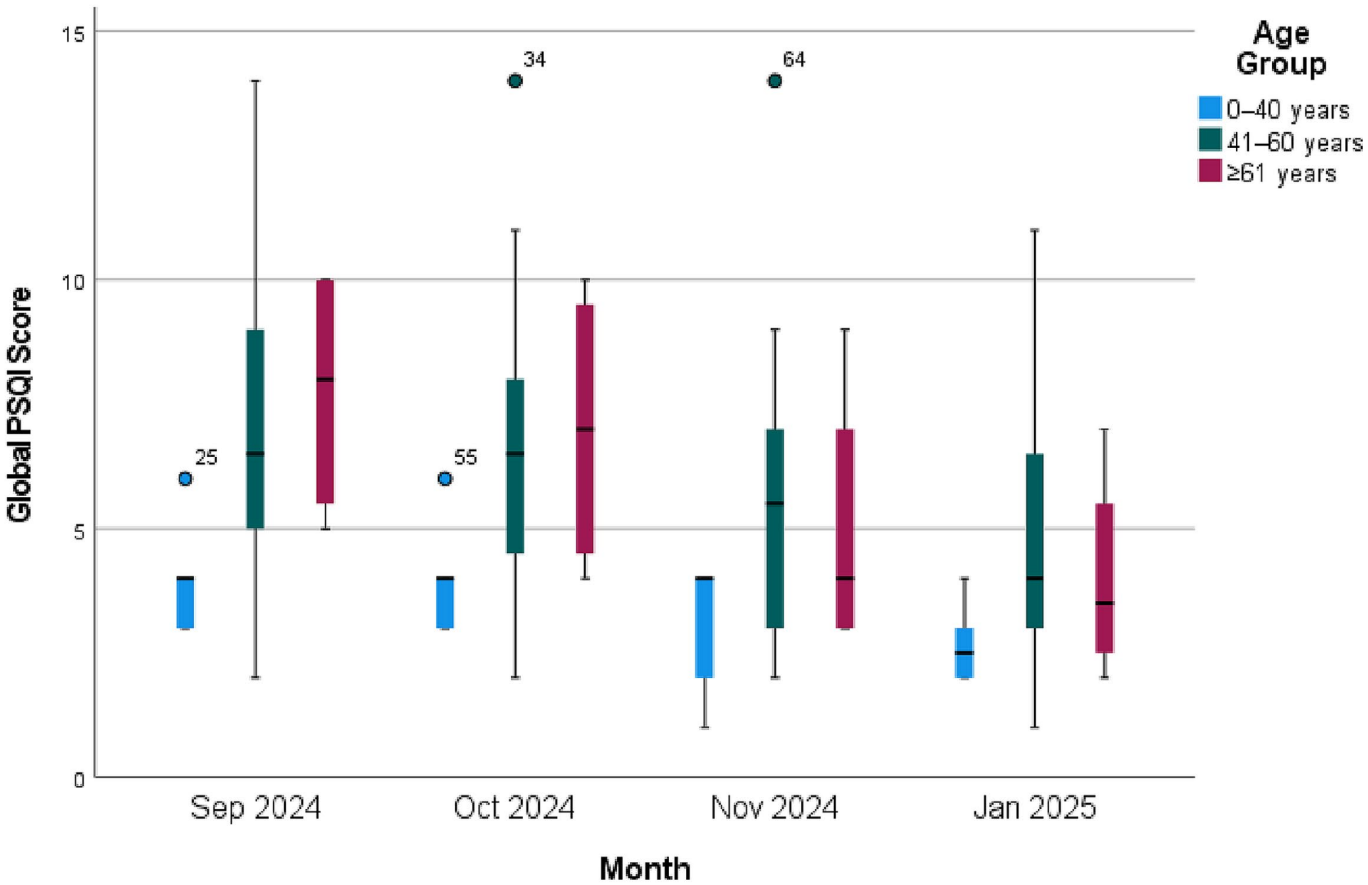
Clustered boxplot of Pittsburgh sleep quality (PSQI) index global scores of 30 participants in 3 private residential care homes for persons with disabilities (PRCHDs) by month by age group, Hong Kong, 2024–2025.

A high proportion of participants residing in PRCHDs experienced poor sleep quality over the 4 months, ranging from 30 to 73%, as determined by the PSQI cut-off score of 5 (Figure 3 and Supplementary Table S2). By January 2025, the average global PSQI score decreased from 7.03 in September 2024 to 5.27 in January 2025, with a narrower range of 1–11, indicating overall improved sleep quality from summer to winter season. Older participants (aged over 60) generally had poorer sleep quality compared to younger participants (aged 40 or below). However, the degree of improvement in PSQI scores over time appears to be consistent across age groups, suggesting that environmental factors, such as temperature and RH in this study, may affect participants of all ages.

Since the global PSQI scores were highly skewed (Skewness = 0.903), which may be due to the high proportion of participants scoring high resulting ceiling effect, the Kruskal-Wallis test was used to compare and analyze the 4 PSQI score medians. A significant difference was found among 4 assessment periods (Kruskal–Wallis *H* = 15.889, *p* = 0.001). To further investigate the difference between months, the Mann–Whitney Test was conducted. As a result, significant differences were found between September and November 2024 (*p* = 0.028), September 2024 and January 2025 (*p* = 0.001), and October 2024 and January 2025 (*p* = 0.002) (Table 2).

**Table 2:** Comparing Pittsburgh sleep quality index (PSQI) global scores of 30 participants in 3 private residential care homes for persons with disabilities (PRCHDs) over 4 months and between every 2 months, Hong Kong, 2024–2025.

Analysis	Kruskal–Wallis test^a^	Mann–Whitney test^b^					
		Sep-Oct	Sep–Nov	Sep–Jan	Oct-Nov	Oct–Jan	Nov–Jan
Chi-square	8.482						
Mann–Whitney *U*		417.5	302	222	331.5	241.5	352.5
95%CI	0.001–0.065	0.476–0.724	0.000–0.049	0.000–0.049	0.024–0.176	0.000–0.049	0.047–0.219
*p*-value	0.037	0.628	0.028	<0.001	0.077	0.002	0.144

^a^Comparing PSQI scores of 30 participants among 4 months (Sep 2024, Oct 2024, Nov 2024, and Jan 2025).

^b^Comparing PSQI scores of 30 participants between 2 months (Sep: Sep 2024, Oct: Oct 2024, Nov: Nov 2024, and Jan: Jan 2025).

### Correlation analysis

For better illustration, we conducted Spearman correlation to analyze the relationship between the means of bedroom temperature and relative humidity and the corresponding PSQI global scores. Significant but rather weak correlations were observed between mean temperature and PSQI scores (*r* = 0.379, *p* < 0.001), which might be acceptable considering the highly skewed distribution of global PSQI scores, and other confounders were not considered in our exploratory study. However, statically significant correlation between mean humidity and PSQI scores could not be found (*r* = 0.167, *p* = 0.068) (Table 3).

**Table 3:** Correlation between bedroom mean temperature, relative humidity, and Pittsburgh sleep quality index (PSQI) global scores of 30 participants in 3 private residential care homes for persons with disabilities (PRCHDs), Hong Kong, 2024–2025.

Analysis	Outcomes	Coefficients	Global PSQI score	Bedroom mean temp in sleeping period (°C)	Bedroom mean RH in sleeping period (%)
Spearman’s rho	Global PSQI score	Correlation Coefficient	1.000	0.379**	0.167
		Sig. (2-tailed)		<0.001	0.068
		*N*	120	120	120
	Bedroom mean temp in sleeping period (°C)	Correlation Coefficient	0.379**	1.000	0.408**
		Sig. (2-tailed)	<0.001		0.000
		*N*	120	120	120
	Bedroom mean RH in sleeping period (%)	Correlation Coefficient	0.167	0.408**	1.000
		Sig. (2-tailed)	0.068	<0.001	
		*N*	120	120	120

**Correlation is significant at the 0.01 level (2-tailed).

GEE were applied in the study to further analyze the PSQI scores with factors that may correlate. Participants’ daily routines, including the hours spent on outdoor activities each week and their sleeping patterns, as well as their self-perceived temperature and humidity levels, are collected through a questionnaire (Supplementary Table S3). Setting subject ID as subject variables, month as within-subject variables, and global PSQI scores as dependent variables, model-based estimator and unstructured were selected for the matrix, considering the limited sample size and no strong assumption about the correlation structure, respectively. A significant correlation was found between PSQI scores and month, implying the changes over time. In order to further investigate the association, all collected factors were calculated with month as the main predictor and model effect (Table 4).

**Table 4:** Generalized estimating equations result of Pittsburgh sleep quality index (PSQI) global scores with demographic, social, and environmental factors, Hong Kong, 2024–2025.

Characteristics	Model^a^		Final model	
	*B*	Sig.	*B*	Sig.
Month				
Sep 2024	2.433	0.000	−4.626	0.000
Oct 2024	2.100	0.000	−2.922	0.000
Nov 2024	0.900	<0.001	−4.401	0.000
Jan 2025	0^a^		0^a^	
Gender				
Female	0.870	0.367		
Male	0^a^			
Age	0.065	0.079		
Diagnosis^b^				
Mental illness^c^	0.176	0.912		
Intellectual disability	−1.187	0.485		
Physical disability	0^a^			
Use of antipsychotics				
Use of antipsychotic	−1.230	0.183		
No use of antipsychotic	0^a^			
Private residential care homes for persons with disabilities (PRCHD)				
PRCHD 1	0.171	0.886		
PRCHD 2	0.201	0.866		
PRCHD 3	0^a^			
Sleeping period				
22:00 to 07:00	1.012	0.010	−0.324	0.000
22:00 to 08:00	1.963	0.238	1.986	0.421
23:00 to 07:00	1.130	0.007	−0.280	<0.001
23:00 to 08:00	0^a^	.	0^a^	
Average hours spent outdoors in last week (hours)	0.188	<0.001	0.197	0.000
Dining area environment				
Minimum temperature (°C)	0.000	0.996		
Maximum temperature (°C)	0.181	0.030		
Mean temperature (°C)	0.204	0.004	0.377	0.000
Standard deviation of temperature (°C)	−0.721	0.107		
Minimum RH (%)	−0.008	0.425		
Maximum RH (%)	−0.063	0.008	0.157	0.000
Mean RH (%)	−0.118	0.030		
Bedroom environment during sleeping period				
Minimum temperature (°C)	0.004	0.908		
Maximum temperature (°C)	0.114	0.006	0.209	0.000
Mean temperature (°C)	0.171	0.023		
Standard deviation of temperature (°C)	0.410	0.007		
Minimum RH (%)	−0.005	0.632		
Maximum RH (%)	0.003	0.672		
Mean RH (%)	0.003	0.744		
Self-perceived scores				
Temperature	0.168	0.068		
Humidity	0.493	<0.001	0.345	0.000

^a^Month is used for the main predictors and model effect, compared with other factors and covariates, as the result is always significant with month (*p* < 0.001).

^b^Diagnosis determined by the diagnosis that affects the participant the most.

^c^Mental illness included schizophrenia and bipolar affective disorder; physical disability means stroke in this study.

Among all collected factors, the PSQI scores significantly correlated with sleeping period (*B* = 1.012–1.963, *p* = 0.007–0.238), hours spent outdoors in a week of participants (*B* = 0.188, *p* < 0.001), and self-perceived humidity level (*B* = 0.493, *p* < 0.001). Dining area and living room environments also showed correlations with the scores. In the dining area, maximum (*B* = 0.191, *p* = 0.030) and mean temperature (*B* = 0.204, *p* = 0.004), and maximum (−0.063, *p* = 0.008) and mean RH (*B* = −0.118, *p* = 0.030) were greatly associated with the scores. Regarding bedroom environment, maximum temperature (*B* = 0.114, *p* = 0.006), and standard deviation of temperature (*B* = 0.410, *p* = 0.007) were correlated to the result. Nevertheless, other factors such as gender, age, and diagnosis did not significantly correlate with the PSQI scores. The final model was conducted with all significant factors, with *p* = 0.000.

## Discussion

In this study, we monitored the indoor temperature and relative humidity in the dining area and bedroom of PRCHDs. The results and corresponding time series indicate that indoor temperatures followed similar seasonal trends to ambient temperatures, particularly during the summer. However, indoor temperatures tended to remain consistently higher than outdoor temperatures after October, likely due to building insulation and limited ventilation during the winter months. This suggests that while indoor temperatures reflect the general seasonal pattern of outdoor conditions, they do not fully replicate them. Additionally, the fluctuation in temperature on the same day was also remarkable, as demonstrated by the range between the maximum and minimum temperatures and the standard deviation of indoor temperature. It was likely related to the use of air-conditioners at night. However, ambient and indoor RH did not show an obvious relationship in this study, which might be attributed to the use of air-conditioners, even for a limited period, and the construction material of the buildings.

It is known that ambient temperature is one of the factors affecting sleep quality (24–26), and sleep quality is essential for mental health, especially for those with mental health issues (21–23). We are therefore concerned about the high ambient temperature in Hong Kong and the accommodation, mainly for people with mental illness. Care home residents with disabilities are assumed to be greatly affected by the condition, as many of them spend most of their time indoors owing to a lack of physical or psychological capacity for outdoor activity, and air-conditioners were only turned on for a minimal period in most PRCHDs. Unfortunately, a notable proportion of participants suffered from poor sleep. In the summer season, over 70% of participants had sleeping problems.

About demographic and social factors, the findings showed that younger care home residents with disabilities and those who spent less time in the PRCHDs usually had better sleep quality. Older age has been associated with poor sleep due to physiological, social, lifestyle, and environmental changes (37, 38). In this study, older participants residing in PRCHDs may be even more sensitive to environmental stressors owing to their mental illness. Additionally, prolonged indoor exposure increases the duration of time spent in high-temperature environments. It may also limit opportunities for physical activity and social interaction, which are important for mental health and sleep quality, especially for the older adult (39, 40). The significant association between indoor temperature and PSQI scores, as demonstrated by the GEE analysis, suggests that higher indoor temperatures were associated with worse sleep quality among PRCHD residents. In this study, diurnal temperature variation was associated with higher PSQI scores. It aligns with the study stating that compress and extreme diurnal temperature variations affect sleep quality (41). Furthermore, indoor RH in the dining areas correlated with sleeping. Considering the result was only found in dining rooms instead of bedrooms, it may imply that even limited use of an air-conditioner might have mitigated the effects of indoor RH in bedrooms during the sleeping period. To our knowledge, no study has investigated the impact of indoor environmental conditions in private residential homes on sleep quality. This study thus offers valuable insights into environmental conditions and their impact on health. The findings could inform future improvements in the environments of PRCHDs to enhance the well-being of residents with disabilities in Hong Kong.

### Limitations and recommendations

The first limitation is that the sample size was relatively small and non-randomized, so the findings may not be generalizable to the entire population, which consists of nearly 4,000 people living in PRCHDs in Hong Kong. However, there were no drop-outs throughout the study. Second, the sleep quality assessment primarily relied on self-reported data, which may be subject to bias, especially for those with intellectual disability (40%), who may find some difficulties in interpreting and answering. Regarding the use of simplified verbal explanations for participants with intellectual disabilities, we acknowledged the potential limitation of this adaptation, noting that it may have introduced variability in how participants understood and responded to the PSQI items. However, we highlighted that this approach was necessary to enable participation by individuals with intellectual disabilities, who are often excluded from similar studies. Additionally, accumulating evidence suggests that, when appropriately adapted and delivered via structured interview, self-report measures of subjective health and quality of life can yield reliable data among adults with mild-to-moderate intellectual disability, particularly when administered by trained staff who know the participants and follow standardized procedures (42) Third, the study did not account for other potential confounding factors, such as noise levels, lighting, and indoor activities, which could also affect sleep quality (43, 44). Our findings should therefore be interpreted as reflecting associations between indoor temperature, humidity, and sleep quality conditional on the measured behavioral and demographic factors, but not fully adjusted for other important aspects of the indoor environment and daily routines. Also, antipsychotic drugs are known to have adverse effects on people with schizophrenia (45–47). However, we could not find a significant correlation between antipsychotic drugs and sleep in this study. It may be the result of a regular and stable medication use pattern among our participants, which did not induce a large effect on their sleep during the study period.

The results of this study have several implications for improving the living conditions in PRCHDs. First, the large proportion of care home residents with disabilities with poor sleep habits needs to be concerned about their higher sensitivity to sleep-related issues, especially in the summer. Additionally, regulating indoor temperature and relative humidity to improve sleep quality in vulnerable populations is essential. Enhancing ventilation and utilizing air conditioning systems are also crucial. Residential care home owners are therefore encouraged to improve these aspects, and the government is advised to implement better regulations and monitoring in the current environment. For instance, provide funding or subsidies for ventilation and air-conditioning systems. Furthermore, measures such as encouraging outdoor activities could be considered to mitigate the adverse effects of high indoor temperatures on sleep quality.

Future studies should aim to include a larger and more diverse sample of PRCHDs and residents to validate the findings. Causal inference approaches, such as Mendelian randomization, could be considered to explore potential causal relationships between relevant traits using genetic instruments. Moreover, incorporating objective measurements of noise and light, systematic documentation of nighttime activities and room occupancy, and, where possible, more detailed characterization of building design and layout to better disentangle environmental pathways affecting sleep is also suggested. Additionally, longitudinal studies with longer follow-up periods may offer more insights into the long-term effects on residents’ health and well-being. Including objective indicators of sleep quality, like actigraphy, may also improve the accuracy of the findings. The cultural or behavioral factors may also be worth elaborating on when considering the low outdoor and social activity level of residents in PRCHDs.

## Conclusion

This study focuses on the impact of indoor temperature and relative humidity on residents’ sleep quality in Hong Kong’s PRCHDs. The results highlight the poor sleep quality experienced by this population, which is significantly influenced by indoor temperature, underscoring the need for environmental modifications to enhance sleep quality and overall well-being for this vulnerable group. It is also recommended that future studies concentrate on larger and more diverse samples to confirm these results and provide evidence-based interventions.

## Data Availability

The original contributions presented in the study are included in the article/Supplementary material, further inquiries can be directed to the corresponding author.
