# Supplementary material for: Investigating the relationship of indoor temperature and humidity with sleeping quality in private residential care homes for persons with disabilities in Hong Kong
Source: Front Public Health. 2026 Feb 23;14:1748619. doi: 10.3389/fpubh.2026.1748619 (PMC12968185; doi:10.3389/fpubh.2026.1748619)

S1 Fig: Indoor and ambient temperature in 3 private residential care homes for persons with disabilities (PRCHDs) from 8/2024 to 2/2025, Hong Kong

a: Indoor and ambient temperature in dining area of PRCHD 1 from 8/2024 – 2/2025

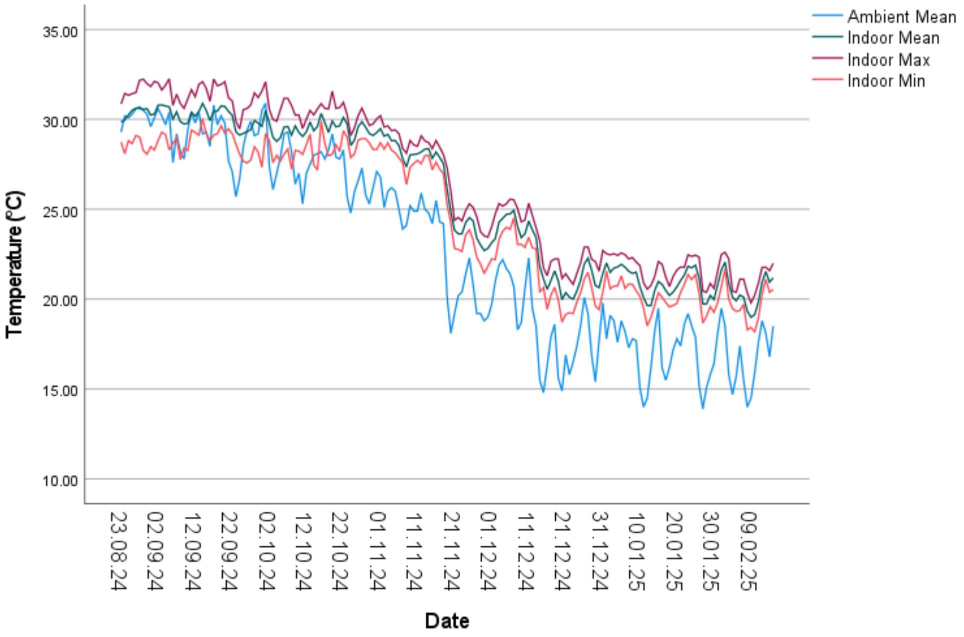

b: Indoor and ambient temperature in bedroom 1 of PRCHD 1 from 8/2024 – 2/2025

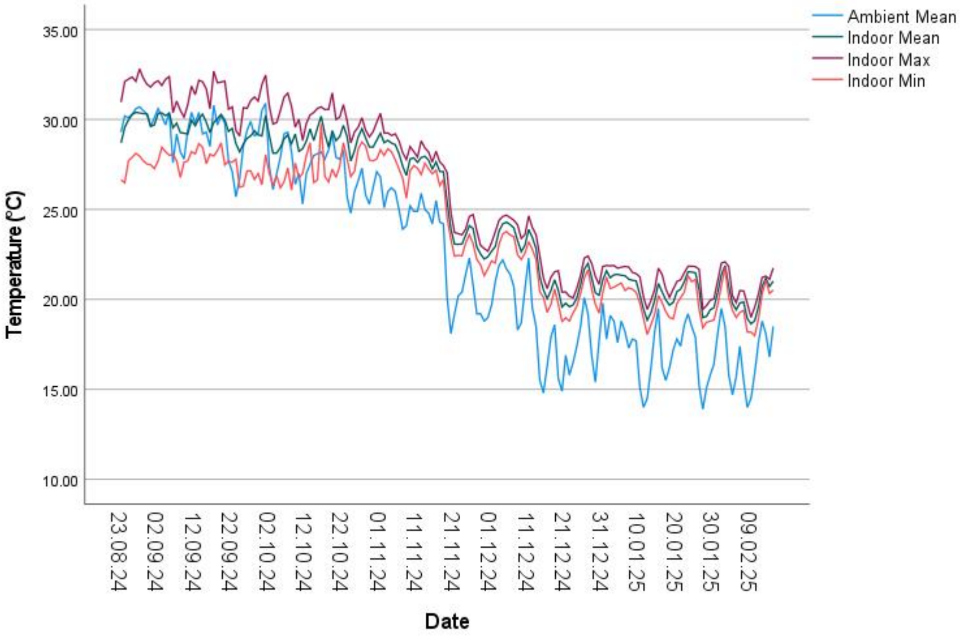

c: Indoor and ambient temperature in bedroom 2 of PRCHD 1 from 8/2024 – 2/2025

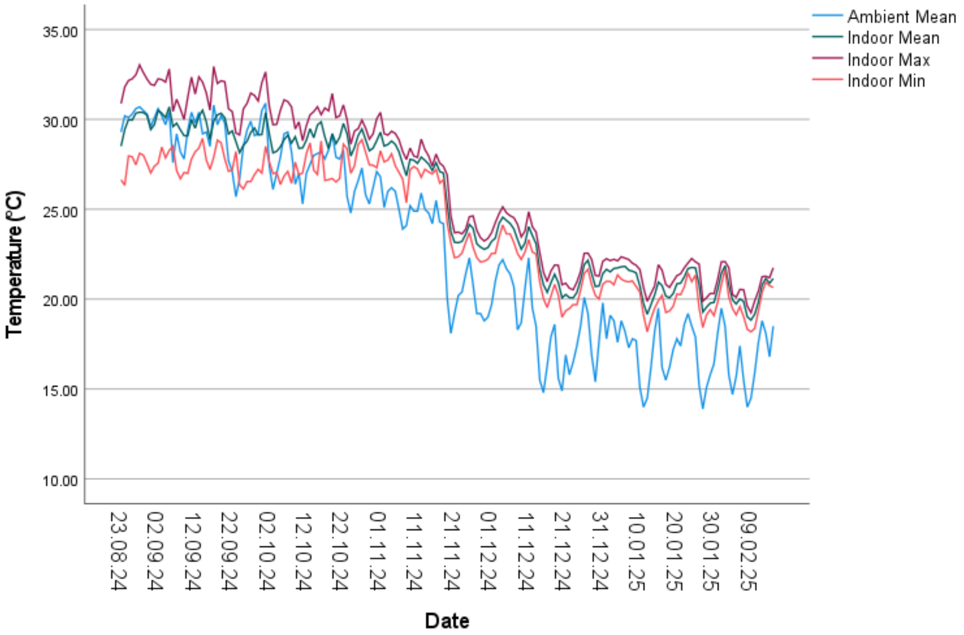

d: Indoor and ambient temperature in dining area of PRCHD 2 from 8/2024 – 2/2025

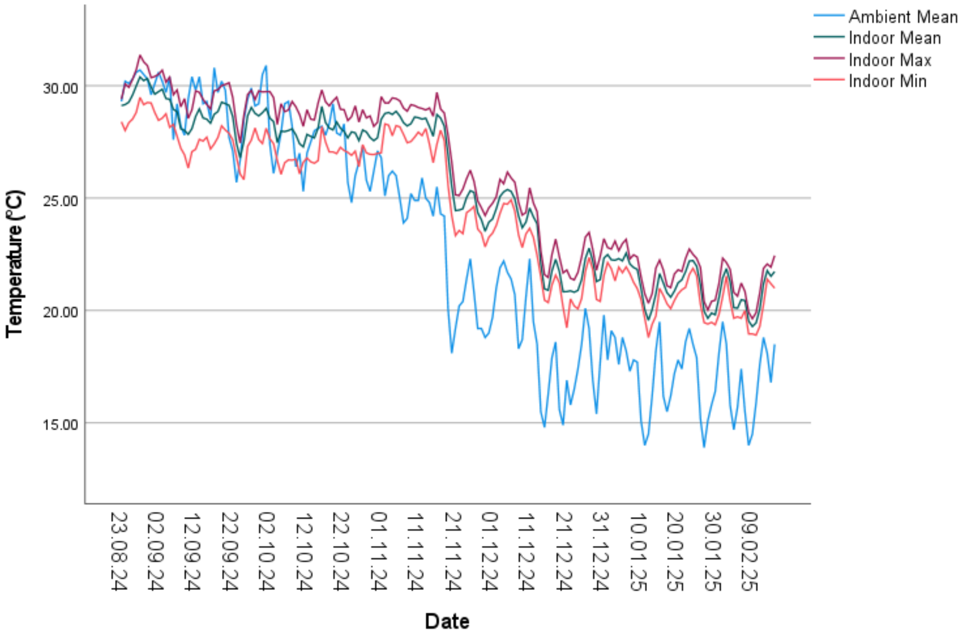

e: Indoor and ambient temperature in bedroom 1 of PRCHD 2 from 8/2024 – 2/2025

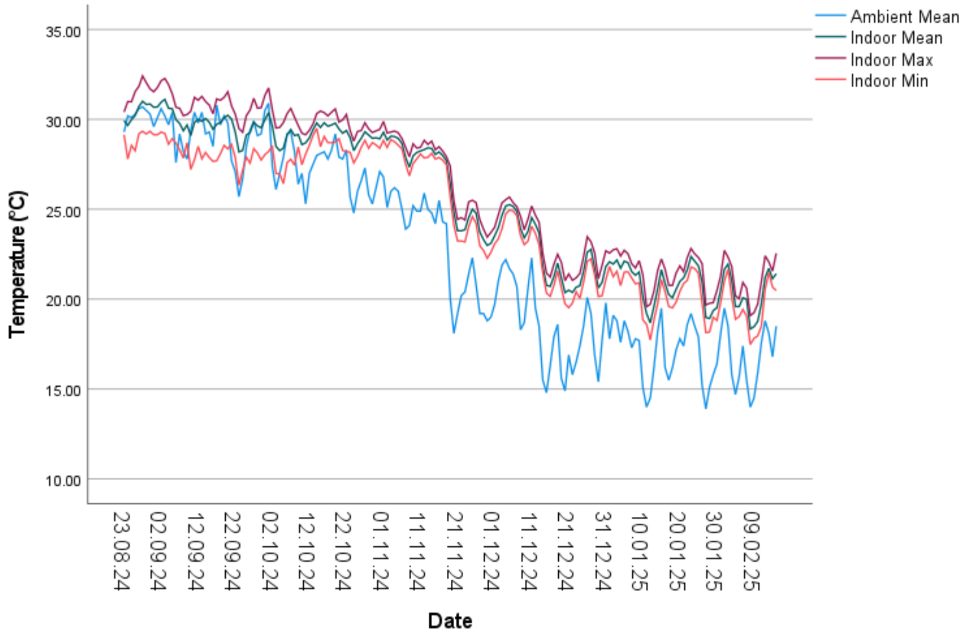

f: Indoor and ambient temperature in bedroom 2 of PRCHD 2 from 8/2024 – 2/2025

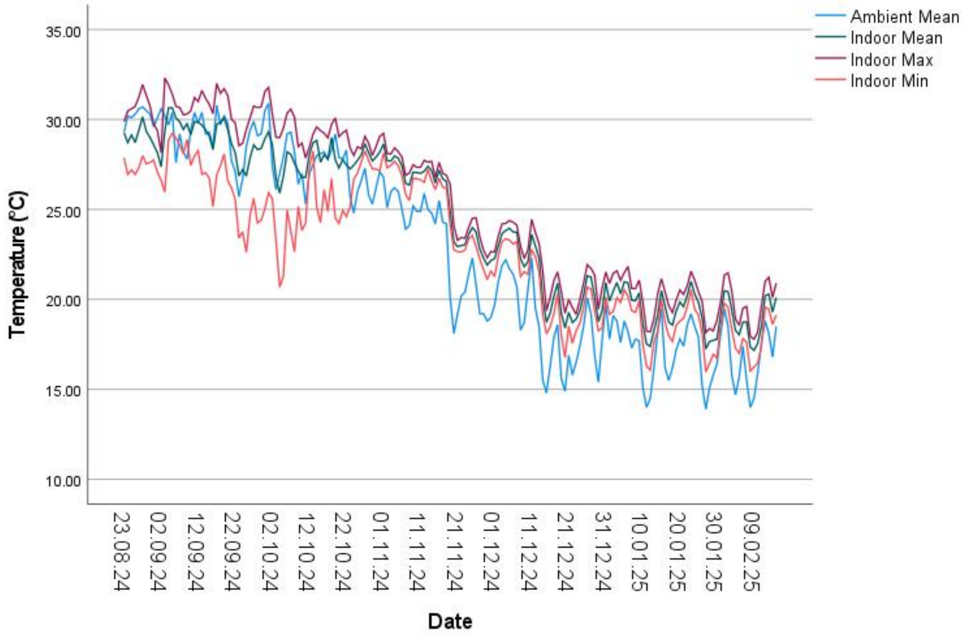

g: Indoor and ambient temperature in dining area of PRCHD 3 from 8/2024 – 2/2025

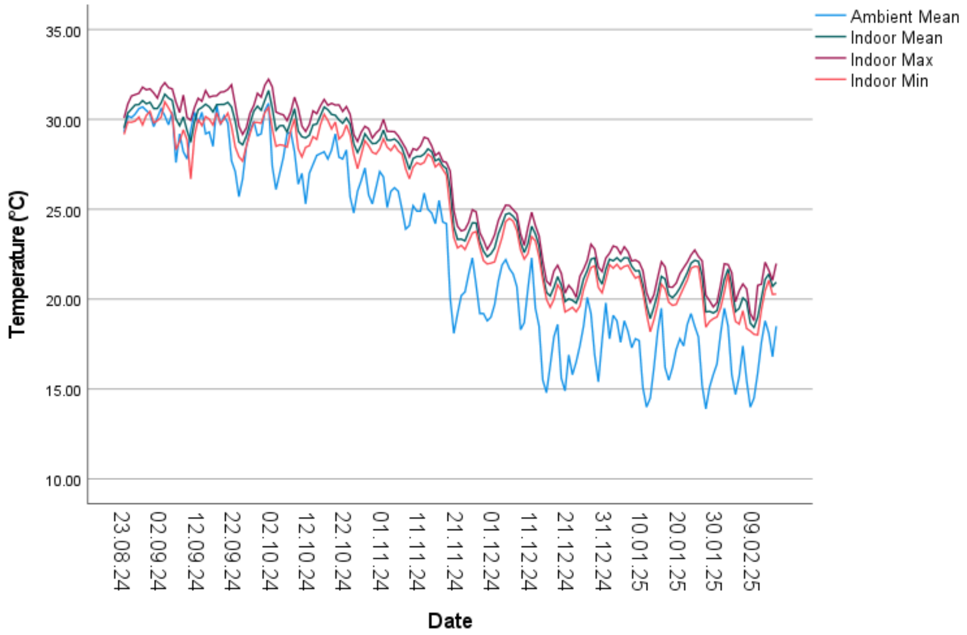

h: Indoor and ambient temperature in bedroom 2 of PRCHD 3 from 8/2024 – 2/2025

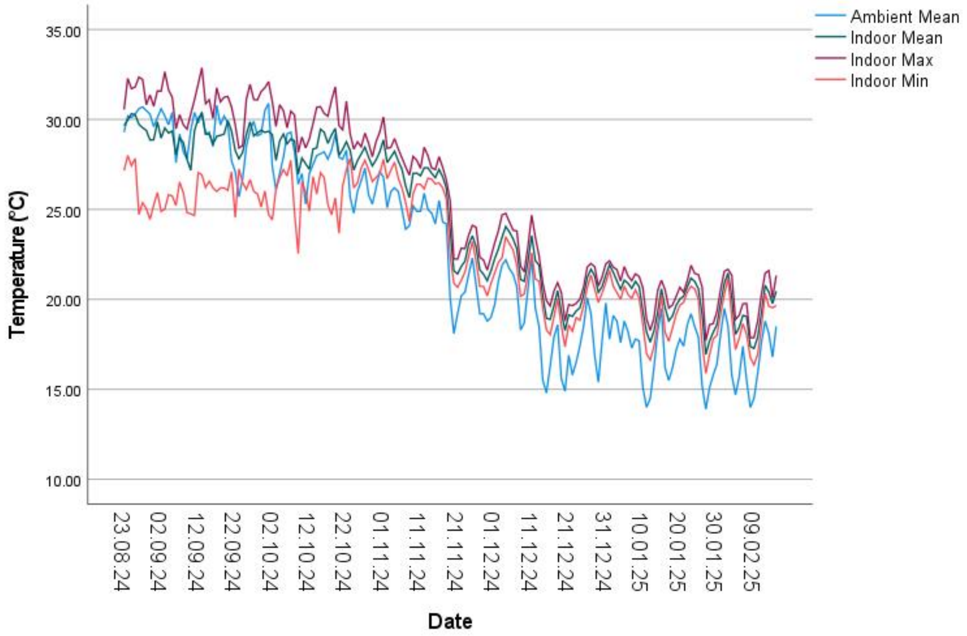

Supplement: Supplementary file 1 [file Data_Sheet_1.pdf]
