# Supplementary material for: Investigating the relationship of indoor temperature and humidity with sleeping quality in private residential care homes for persons with disabilities in Hong Kong
Source: Front Public Health. 2026 Feb 23;14:1748619. doi: 10.3389/fpubh.2026.1748619 (PMC12968185; doi:10.3389/fpubh.2026.1748619)
Supplement: Supplementary file 2 [file Data_Sheet_2.pdf]

S1 Questionnaire: Questionnaire for face-to-face survey

Question	Answer
Age	_____(DOB)
Major Diagnosis	Physical: Stroke/ Schizophrenia/ Mood disorder/Intellectual disability (Mild/Moderate/Severe)/ Other issue
Other medical conditions required long-term medication and/or regular medical follow-ups	Heart disease/ Diabetes/ Other issue
Sleeping-related medication in the last week	Yes<br>No
Time spent in the dining area every day<br>(Select period)	06:00-08:00<br>08:00-10:00<br>10:00-12:00<br>12:00-14:00<br>14:00-16:00<br>16:00-18:00
Time spent in the bedroom every day<br>(Select period)	06:00-08:00<br>08:00-10:00<br>10:00-12:00<br>12:00-14:00<br>14:00-16:00<br>16:00-18:00<br>18:00-06:00
Outdoor activity frequency in last week	_____Days per week
Time spent on outdoor activity in the last week<br>(Select period)	06:00-08:00<br>08:00-10:00<br>10:00-12:00<br>12:00-14:00<br>14:00-16:00
Overall self-perceived temperature in last week	Very cold (1)<br>Cold (2)<br>Neutral (3)<br>Hot (4)<br>Very hot (5)
Overall self-perceived humidity in last week	Very dry (1)<br>Dry (2)<br>Neutral (3)<br>Wet (4)<br>Very wet (5)
