# Supplementary material for: Investigating the relationship of indoor temperature and humidity with sleeping quality in private residential care homes for persons with disabilities in Hong Kong
Source: Front Public Health. 2026 Feb 23;14:1748619. doi: 10.3389/fpubh.2026.1748619 (PMC12968185; doi:10.3389/fpubh.2026.1748619)
Supplement: Supplementary file 3 [file Data_Sheet_3.pdf]

S1 Table: Environmental characteristics of 3 private residential care homes for persons with disabilities (PRCHDs) in Hong Kong, 2024-2025

PRCHD	Total area (square feet)	Floor Height (meter)	Residents numbers	Windows number per 1000 square feet	Fans number per 1000 square feet	Air-conditioning	Air-conditioner number per 1000 square feet	Air-conditioning in dining area	Air-conditioning in bedroom
1	6000	2.9	85	8.33	Ceiling fan: 2.66	Yes	3.83	11:00-13:00	19:00-06:00
2	3400	5.0	52	11.76	Ceiling fan: 5.29<br>Wall mount fan: 8.82	Yes	3.53	No	19:00-06:00
3	1700	3.0	26	9.41	Wall mount fan: 3.53	Yes	2.35	07:00-12:00<br>16:00-18:00	19:00-06:00
