# Supplementary material for: Investigating the relationship of indoor temperature and humidity with sleeping quality in private residential care homes for persons with disabilities in Hong Kong
Source: Front Public Health. 2026 Feb 23;14:1748619. doi: 10.3389/fpubh.2026.1748619 (PMC12968185; doi:10.3389/fpubh.2026.1748619)
Supplement: Supplementary file 5 [file Data_Sheet_5.pdf]

S2 Table: Pittsburgh sleep quality (PSQI) index global scores of 30 participants in 3 private residential care homes for persons with disabilities (PRCHDs) over 5 months, Hong Kong, 2024-2025.

				PSQI global score			
				Sep-24	Oct-24	Nov-24	Jan-25
Participant	Age	Diagnosis [a][b]	PRCHD				
1	69	Stroke	1	10	10	9	7
2	54	Schizophrenia	1	6	6	3	2
3	57	Stroke	1	5	4	3	3
4	55	BAD	1	14	14	14	11
5	58	Schizophrenia	1	8	7	6	3
6	35	Mild ID	1	4	4	4	3
7	46	Schizophrenia	1	7	7	5	4
8	52	Mild ID	1	6	6	4	3
9	33	Schizophrenia	1	4	4	4	2
10	64	Schizophrenia	1	6	4	3	2
11	57	Schizophrenia	2	4	4	3	2
12	55	Schizophrenia	2	11	11	8	8
13	50	Schizophrenia	2	3	3	2	1
14	47	Schizophrenia	2	7	7	6	4
15	46	Schizophrenia	2	11	11	8	7
16	55	Schizophrenia	2	5	5	7	6
17	56	Stroke	2	5	4	3	3
18	52	Dementia	2	2	2	2	2
19	52	Dementia	2	10	10	9	7
20	43	Schizophrenia	2	8	7	6	5
21	66	Schizophrenia	3	10	9	5	4
22	59	Mild ID	3	6	5	4	4
23	34	Mod ID	3	4	4	4	4
24	18	Mod ID	3	3	3	1	2

25	22	Mild ID	3	6	6	4	3
26	41	Mod ID	3	9	8	7	7
27	71	Mild ID	3	5	5	3	3
28	36	Mild ID	3	3	3	2	2
29	53	Schizophrenia	3	6	6	5	4
30	48	Schizophrenia	3	9	8	7	6

[a] Diagnosis determined by the diagnosis that affects the participant the most

[b] BAD = Bipolar affective disorder, ID = Intellectual disability, Mod ID = Moderate intellectual disabilities
