# Supplementary material for: Investigating the relationship of indoor temperature and humidity with sleeping quality in private residential care homes for persons with disabilities in Hong Kong
Source: Front Public Health. 2026 Feb 23;14:1748619. doi: 10.3389/fpubh.2026.1748619 (PMC12968185; doi:10.3389/fpubh.2026.1748619)
Supplement: Supplementary file 6 [file Data_Sheet_6.pdf]

S3 Table: Questionnaire results of 30 participants in 3 private residential care homes for persons with disabilities (PRCHDs) over 4 months, Hong Kong, 2024-2025

	Use of antipsychotics				Hours spent in the dining area in daytime				Hours spent in the bedroom in daytime				Sleeping period [a]				Hours of outdoor activities per week				Self-perceived temperature level [b]				Self-perceived humidity level [c]			
	Sep -24	Oct -24	Nov -24	Jan -25	Sep -24	Oct -24	Nov -24	Jan -25	Sep -24	Oct -24	Nov -24	Jan -25	Sep -24	Oct -24	Nov -24	Jan -25	Sep -24	Oct -24	Nov -24	Jan -25	Sep -24	Oct -24	Nov -24	Jan -25	Sep -24	Oct -24	Nov -24	Jan -25
**1**					6	6	6	6	18	18	18	18	A	A	A	A	0	0	0	0	4	4	4	1	5	4	3	2
**2**	Yes	Yes	Yes	Yes	8	8	8	8	14	14	14	14	C	C	A	A	6	6	6	4	5	5	4	2	5	5	3	3
**3**					4	4	4	4	20	20	20	20	C	C	C	A	0	0	0	0	4	5	3	2	3	3	3	2
**4**	Yes	Yes	Yes	Yes	8	8	6	8	16	16	18	16	A	A	A	A	0	0	0	0	3	3	3	3	3	3	3	3
**5**	Yes	Yes	Yes	Yes	4	4	4	4	20	20	20	20	C	C	C	C	0	0	0	0	3	3	3	3	4	3	3	3
**6**					6	6	6	6	18	18	18	18	C	C	C	C	2	2	2	2	5	4	4	2	3	4	4	3
**7**	Yes	Yes	Yes	Yes	6	6	6	6	18	18	18	18	D	D	D	D	4	4	4	4	4	4	3	2	4	3	3	1
**8**					6	6	6	6	18	18	18	18	B	B	B	B	0	0	0	0	4	4	3	2	4	4	3	1
**9**	Yes	Yes	Yes	Yes	6	6	6	6	12	12	12	12	D	D	D	D	20	20	20	20	4	4	4	1	3	3	3	2
**10**	Yes	Yes	Yes	Yes	6	6	8	6	18	18	16	16	C	C	C	A	5	5	5	5	5	4	4	2	5	4	4	3
**11**	Yes	Yes	Yes	Yes	6	6	6	6	18	18	18	18	D	D	D	D	3	3	4	3	4	4	3	2	3	3	3	2
**12**	Yes	Yes	Yes	Yes	4	4	4	4	18	18	18	18	A	A	A	A	0	0	0	0	3	3	3	1	3	3	3	1
**13**	Yes	Yes	Yes	Yes	6	6	6	6	18	18	18	18	A	A	C	C	2	2	2	2	4	4	3	2	4	4	4	3
**14**	Yes	Yes	Yes	Yes	4	4	4	4	20	20	20	20	A	A	A	A	0	0	0	0	4	4	4	2	5	4	4	3
**15**	Yes	Yes	Yes	Yes	4	4	4	4	20	20	20	20	D	D	D	D	4	4	4	6	5	4	4	2	5	5	4	3
**16**	Yes	Yes	Yes	Yes	4	4	4	2	20	20	16	18	C	C	C	D	9	9	24	20	4	4	3	2	4	4	4	3
**17**					4	4	4	4	20	20	20	20	C	C	C	C	4	4	6	4	5	5	4	2	5	5	4	2

**18**					6	6	6	6	18	18	18	18	A	A	A	A	0	0	0	0	4	3	3	2	3	3	3	3
**19**					4	4	4	4	20	20	20	20	C	C	C	C	0	0	0	0	4	4	4	2	4	4	4	3
**20**	Yes	Yes	Yes	Yes	4	4	4	4	20	20	20	20	B	B	B	B	0	0	0	0	3	3	3	1	3	3	3	3
**21**	Yes	Yes	Yes	Yes	6	6	6	6	18	18	18	18	A	A	A	A	0	0	2	0	5	5	4	3	4	4	4	3
**22**					8	8	8	8	10	10	10	10	C	C	C	C	30	30	30	30	4	4	3	2	5	4	3	3
**23**					8	8	8	8	10	10	10	10	C	C	C	C	30	30	30	30	4	4	4	3	4	4	4	3
**24**					8	8	8	8	10	10	10	10	A	A	D	D	30	30	30	30	3	3	3	1	3	3	3	2
**25**					10	10	10	10	14	14	14	14	D	D	D	D	0	0	0	0	4	4	4	1	4	3	3	1
**26**					6	6	6	6	18	18	18	18	A	A	A	A	0	0	0	0	5	5	4	2	4	4	4	3
**27**					8	8	8	8	16	16	16	16	A	A	A	A	0	0	0	0	5	4	3	2	5	4	3	3
**28**					8	8	8	8	10	10	10	10	C	C	C	C	30	30	30	30	4	5	4	2	4	4	4	3
**29**	Yes	Yes	Yes	Yes	4	4	4	4	20	20	20	20	C	C	C	C	2	2	2	2	5	5	4	2	4	4	4	3
**30**	Yes	Yes	Yes	Yes	6	6	8	6	18	18	16	18	B	B	B	B	2	0	2	0	4	4	4	2	4	4	4	2

[a] Sleeping pattern: A=22:00 to 07:00, B=22:00 to 08:00, C=23:00 to 07:00, D=23:00 to 08:00

[b] Self-perceived temperature level: 1=Very cold, 2=cold, 3=Neutral, 4=Hot, 5=Very hot

[c] Self-perceived humidity level: 1=Very dry, 2=Dry, 3=Neutral, 4=Wet, 5=Very wet
